# Diagnosis and management of primary hyperaldosteronism in patients with hypertension: a practical approach endorsed by the British and Irish Hypertension Society

**DOI:** 10.1038/s41371-023-00875-1

**Published:** 2023-11-14

**Authors:** Luca Faconti, Spoorthy Kulkarni, Christian Delles, Vikas Kapil, Philip Lewis, Mark Glover, Thomas M. MacDonald, Ian B. Wilkinson

**Affiliations:** 1 King’s College London British Heart Foundation Centre, Department of Clinical Pharmacology, 4th Floor, North Wing, St. Thomas’ Hospital, Westminster Bridge, London, SE17EH UK; 2 Cambridge University hospitals NHS foundation trust, Cambridge United Kingdom (S.K.), Cambridge, UK; 3 School of Cardiovascular and Metabolic Health, University of Glasgow, Glasgow, G12 8TA UK; 4 William Harvey Research Institute, Centre for Cardiovascular Medicine and Devices, Queen Mary University London, London, EC1M 6BQ UK; 5 Barts BP Centre of Excellence, Barts Heart Centre, London, EC1A 7BE UK; 6 Department of Cardiology, Stockport NHS Foundation Trust, Stockport, UK; 7 Deceased, formerly Division of Therapeutics and Molecular Medicine, School of Medicine, University of Nottingham, Nottingham, UK; 8 Division of Molecular and Clinical Medicine, University of Dundee, Dundee, UK; 9 Division of Experimental Medicine and Immunotherapeutics, Department of Medicine, University of Cambridge, Cambridge, UK

**Keywords:** Adrenal gland diseases, Hypertension

## Abstract

Alongside the lack of homogeneity among international guidelines and consensus documents on primary hyperaldosteronism, the National UK guidelines on hypertension do not provide extensive recommendations regarding the diagnosis and management of this condition. Local guidelines vary from area to area, and this is reflected in the current clinical practice in the UK. In an attempt to provide support to the clinicians involved in the screening of subjects with hypertension and clinical management of suspected cases of primary hyperaldosteronism the following document has been prepared on the behalf of the BIHS Guidelines and Information Service Standing Committee. Through remote video conferences, the authors of this document reviewed an initial draft which was then circulated among the BIHS Executive members for feedback. A survey among members of the BIHS was carried out in 2022 to assess screening strategies and clinical management of primary hyperaldosteronism in the different regions of the UK. Feedback and results of the survey were then discussed and incorporated in the final document which was approved by the panel after consensus was achieved considering critical review of existing literature and expert opinions. Grading of recommendations was not performed in light of the limited available data from properly designed randomized controlled trials.

## Introduction

Primary hyperaldosteronism (PA) comprise a heterogeneous group of familial and sporadic disorders characterized by a relatively autonomous renin-independent aldosterone production syndrome [1]. PA can be classified into subtypes, that differ in terms of clinical management, including unilateral forms, which may be treated surgically (such as aldosterone-producing adenomas), and bilateral forms (such as bilateral adrenal hyperplasia/nodules) requiring medical management [1–5]. The classical description of the disorder firstly made by Conn and colleagues in 1955 (syndrome of hypertension, sodium retention, and hypokalemic alkalosis that could be cured by removal of an adrenal cortical tumour [6]) can be found in approximately one third of subjects, whilst adenoma with normokalaemia is the most common presentation [1]. PA is a well characterized form of secondary hypertension in adults [5] particularly in patients with resistant hypertension [7, 8]. Apart from being a potentially treatable form of hypertension, PA may also be an independent cardiovascular risk factor as affected subjects display an increased cardiovascular risk (including stroke, coronary artery disease, atrial fibrillation, heart failure, diabetes and metabolic syndrome), which is not fully explained by blood pressure level [9, 10]. However, uncertainty exists regarding the prevalence of the syndrome in the general hypertensive population due to screening bias and non-uniform criteria used to diagnose [7, 11]. In fact, it is commonly accepted that there is a continuum between low-renin primary (essential) hypertension and PA [12] and a diagnosis is only obtained in patients who fulfil the criteria suggested for its diagnosis. Because of this, PA remains an often unrecognized condition [13, 14] particularly in subjects with resistant hypertension. The aim of this document is therefore to raise awareness about PA, guide clinicians in the screening/diagnosis process and support the treatment strategy to mitigate the adverse cardiometabolic effects in otherwise undiagnosed individuals.

## Prevalence of primary hyperaldosteronism

The prevalence of PA in the hypertensive population remains controversial and depends upon the population being examined [15, 16]. Historically, PA has been considered a very rare disease [17] although often only selected populations were considered (such as patients with hypokalaemia). More recent investigations disputed those early findings [3, 11], although the majority of studies were carried out in specialized referral centres, raising concerns regarding potential selection bias. In fact, the prevalence of PA varies according to the clinical setting and the criteria used for its diagnosis [11, 15, 18].

In the general hypertensive population, the prevalence of PA has significant uncertainties (from almost 6% [11] to less than 2% [19]), with surgically treatable forms representing half of the cases [20]. Data from primary care are in fact conflicting with a significantly higher prevalence being reported [21, 22], although many studies were based upon an elevated aldosterone to renin ratio (ARR) without complete diagnostic work up (ARR is related with false positive results in up to 30–50% of patients who then undergo further confirmatory testing and investigations [23]).

The prevalence of PA increases with the severity of hypertension and in specialist referral centres, the proportion of subjects with PA is likely to be substantially higher compared to the primary care setting [15]. Data from European specialist centres suggests an overall prevalence greater than 5% [3]. It is also commonly accepted that the prevalence of PA is higher in subjects with hypokalaemia and increases with the severity of hypertension [11, 24], thus exceeds 10% in patients with resistant hypertension [7, 8].

Despite the above-mentioned limitations, a large body of evidence over the last few decades has confirmed that PA is less rare than originally thought, although uncertainties regarding the precise prevalence of the condition in unselected hypertensive populations persist. Finally, the prevalence of PA has not been systematically determined amongst different ethnicities including subjects of African origin background. Many studies, including some performed in the UK, have demonstrated that plasma renin level is lower in hypertensive black individuals compared with white subjects [25] although the evidence that aldosterone-associated hypertension is more common in African origin  subjects is lacking [8].

### Summary

- Data on PA prevalence, in unselected hypertensive populations, are conflicting.
- The prevalence of PA increases with the severity of hypertension and is likely to exceed 10% patients with resistant hypertension or those with hypokalaemia.

## Screening strategy for PA in hypertension

In the NICE Clinical Knowledge Summaries (CKS), clinical features of PA include hypokalaemia (spontaneous or thiazide-induced), metabolic alkalosis (elevated serum bicarbonate) and plasma sodium level greater than 140 mmol/L. Symptoms are non-specific, but might very rarely include tetany, muscle weakness, nocturia, or polyuria. Although these features undoubtedly help identify patients who are likely to present with PA, their absence should not exclude patients from being investigated, as most patients with PA are asymptomatic. Moreover, hypokalaemia which was considered a cardinal feature of PA occurs only in a minority of patients [23, 26, 27], and symptoms are even more rare. It is now commonly accepted that PA should not be considered a rare clinical phenotype but a form of hypertension characterized by autonomous aldosterone secretion which can coexist with normokalaemia and morphologically normal adrenal glands [28–31].

Table 1 summarizes the clinical scenarios in which the BIHS recommends diagnostic work-up for suspected PA among patients with arterial hypertension. The list includes three clinical situations more frequently associated with PA, and the screening of young subjects with newly diagnosed hypertension. In the latter group, routine screening for PA can be considered to facilitate an early diagnosis followed by a targeted treatment strategy. Despite most expert guidelines recommending screening for PA in these clinical conditions (such as resistant hypertension), in clinical practice only a fraction of the eligible patients are likely to be evaluated [32]. Some practical guidelines also recommend screening patients with obstructive sleep apnoea (although this indication has been questioned [33, 34]), as well as subjects with evidence of hypertension-mediated organ damage (HMOD) [35–37] “more severe” than expected from severity and duration of hypertension. There is evidence that aldosterone—in the presence of high salt intake [38]—can contribute to left ventricular hypertrophy and cardiac fibrosis [39] through mechanisms that are at least partly independent of its effects on blood pressure [10]. However, no clinically meaningful reference values are available to define this “inappropriate” remodelling. Finally, in the PAPPHY (Prospective Appraisal of the Prevalence of Primary aldosteronism in HYpertensive patients) study, the prevalence of PA amongst patients with atrial fibrillation was almost four-times higher than in resistant hypertension [33, 40]. However, considering that atrial fibrillation is the most common cardiac arrhythmia in the general population (as well as in subjects with hypertension) and that conflicting data exist on the prevalence of PA in this group [41], the cost effectiveness of systematic screening in these patients warrants evaluation.

**Table 1:** Clinical situations in which screening of PA is recommended by the BIHS.

Condition:	Rationale:
Adults with hypertension < 40 years	Specialist evaluation of secondary causes of hypertension to be considered (NICE guidelines 2019 [38]) and higher chance of successful treatment in case of adrenalectomy
Hypertension with spontaneous or thiazide-induced hypokalaemia	Serum potassium level <3.5 mmol/l in absence of other potential causes (such as vomiting, diarrhoea, diabetic ketoacidosis) requires investigations to exclude PA. The prevalence of PA in patients with hypokalaemia has been reported to be 28.1% [39] Hypokalaemia is also more common in patients with aldosterone-producing adenoma compared with idiopathic forms of PA [3].
Resistant hypertension	High prevalence of PA (>10%) [22, 40]
Hypertension associated with adrenal incidentaloma	Prevalence of adrenal incidentalomas is at least 2 % in abdominal CT scans, with most lesions being adrenocortical adenomas [41]. Approximately 1% of the adrenal incidentalomas are aldosterone-producing adenomas [42]. Incidentalomas must be evaluated regarding their functional status and malignant potential. Endocrine review is advisable.

### Overall summary of recommendations for screening strategy in patients with hypertension

- Age of onset <40 years.
- Spontaneous or thiazide-induced hypokalaemia.
- Failure to achieve blood pressure control on three antihypertensive drugs, including a diuretic, i.e. resistant hypertension.
- Co-existing adrenal incidentaloma.

## Diagnostic workup

There are three cardinal pathophysiologic features of PA firstly described by Conn and colleagues [42] that still influence our diagnostic approach:

1. An autonomous aldosterone production suppressing plasma renin.
2. A blunted renin response to stimulation (such as administering an angiotensin-converting enzyme inhibitor (ACE-I)).
3. Lack of aldosterone suppression by volume expansion.

**Fig. 1:**
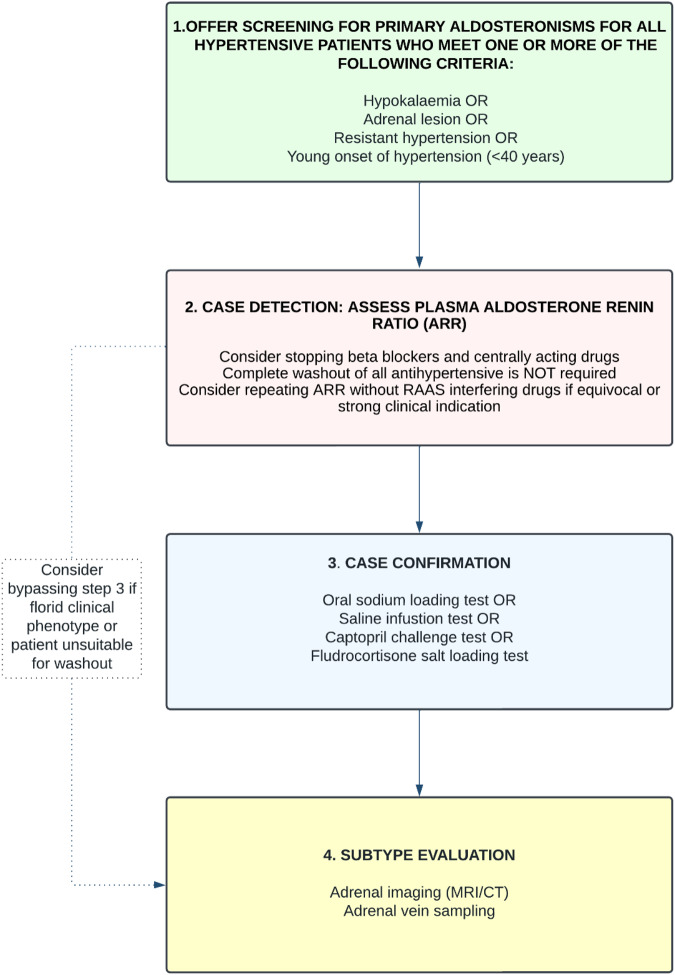
Schematic algorithm for diagnostic workup of primary hyperaldosteronism (PA) in patients with hypertension. ARR aldosterone to renin ratio, RAAS renin-angiotensin-aldosterone system.

The diagnosis of PA requires demonstration of a low or undetectable renin level, plasma aldosterone concentration that is inappropriately high for salt and volume status and a confirmatory test to demonstrate that aldosterone secretion is unresponsive to manoeuvres that perturbate renin production. Thus, the diagnosis of PA usually requires a two-step approach and subtype evaluation (Fig. 1):

1. case detection
2. case confirmation

## Case detection

The most commonly used test for case detection is the ARR. The ARR was introduced in clinical practice to overcome some of the methodological limitations related to the isolated interpretation of plasma aldosterone concentration (and renin) [43]. In well controlled conditions, an elevated ARR had a relatively good sensitivity for diagnosing PA [1] and, as such, is widely used for screening. Several authors have however questions its reliability due to significant variation on repeated measurements [44] and it is now commonly accepted  that the interpretation of the ARR is subject to several pitfalls [45]. In fact, ARR is influenced by electrolyte abnormalities, drugs [1], circadian rhythm [46] and posture. Hypokalaemia, for example, reduces plasma aldosterone concentration and consequentially the ARR. Ideally the ARR should be evaluated in the absence of interfering drugs and in the morning [47]. Non-steroidal anti-inflammatory drugs, steroids, oestrogen-containing medications, liquorice, dopaminergic and antihistaminergic medications and antidepressants all affect both aldosterone and renin levels [48, 49]. Likewise, several drugs including  antihypertensive medicationss alter the ARR (diuretics, ACE-I, angiotensin II receptor blockers (ARBs), beta-blockers and centrally acting drugs) (Table 2). It is commonly accepted that long-acting non-dihydropyridine calcium channel antagonists, doxazosin, hydralazine, and moxonidine minimally affect plasma aldosterone concentration and ARR, and should be considered the best therapeutic options in patients undergoing washout.

**Table 2:** Effects of sodium/potassium loading and drugs on aldosterone concentration (PAC), renin level and aldosterone renin ratio (ARR).

Factors	EFFECT on PAC	Effect on renin	Effect on ARR
Hypokalaemia	↓	→↑	↓
Potassium loading	↑	→↓	↑
Sodium restriction	↑	↑↑	↓
Sodium loading	↓	↓↓	↑
NSAIDs	↓	↓↓	↑
SGLT2-i	→	↑	↓
SSRI	↑	↑↑	↓
Oral contraceptives	↑	↑↓	↑↓
β-Adrenoreceptor antagonists	↓	↓↓	↑
DHP calcium channel blockers	→↓	→	↓
Non-DHP calcium channel blockers	→	→	→
α1-antagonists	→	→	→
Hydralizine	→	→	→
Moxonidine	→	→	→
Clonidine, Alpha-methyldopa	↓	↓↓	↑
ACE-I	↓	↑↑	↓
ARB	↓	↑↑	↓
Direct renin inibithors	↓	↑↓	↑↓
K+ sparing diuretics	↑	↑↑	↓
K+ wasting diuretics	→↑	↑↑	↓

*DHP* dihydropyridine, *ACE-I* angiotensin-converting enzyme inhibitor, *ARB* angiotensin II receptor blocker, *NSAIDs* non-steroidal anti-inflammatory drugs, *SGLT2-i* sodium-glucose co-transporter-2 inhibitor, *SSRI* selective serotonin reuptake inhibitors.

Despite being widely used as a screening test for PA, there are well known limitations to the use and interpretation of ARR in clinical practice. Firstly, pharmacological washout can be impractical for patients and clinicians (especially in patients with resistant hypertension and multiple comorbidities) and may expose the patient to risks related to uncontrolled hypertension. From a methodological aspect, the lack of uniformity in diagnostic protocols and assay methods for measuring the ARR has also been associated with substantial variability in cut-off values (Table 3 [1]). These arbitrary cut-offs values have been criticized [50] and their reliability is still a matter of discussion [51] since ARR can vary from high to normal on repeated studies [52]. Particularly in case of clinical suspicion for PA repeat testing, preferably under optimal conditions—with minimal delay between sampling and lab processing—is advised as sampling and processing errors may have occurred. Similarly, if ARR values are borderline before proceeding for further investigations, a repeated test under standardized conditions is advisable.

**Table 3:** Most commonly adopted cut off values of ARR depending on the assay used and units.

	PRA, ng/mL/h	PRA, pmol/L/min	DRC, mU/L	DRC, ng/L
PAC (as ng/dL)	30	2.5	3.7	5.7
PAC (as pmol/L)	750	60	91	144

Modified by J. W. Funder et al. [1].

The most commonly adopted cut-off values are 30 for PAC and PRA in conventional units (equivalent to 830 when PAC is in SI units) and 750 when PAC is expressed in SI units (equivalent to 27 in conventional units).

*PAC* plasma aldosterone concentration, *PRA* plasma renin activity, *DRC* direct renin concentration, *PRA* plasma renin activity, *DRC* direct renin concentration, *PAC* plasma aldosterone concentration.

Because of the limitations, the BIHS recommends that alongside the ARR, the individual components of the ratio should be considered in the decision making process [53]. Plasma renin can be assessed as activity—by measuring the amount of angiotensin 1 generated over time—or with direct essays that quantify renin mass. In most laboratories the direct active renin concentration has replaced the plasma renin activity because it is cheaper, faster, automatable, and also because it allows samples to be handled at room temperature [54]. Both methodologies, however, lose precision when measuring very low renin values and it has been suggested a low detection limit for the evaluation of ARR is used (e.g. 0.2 ng/mL/h or 2 mIU/L) to avoid false positive results, although this is not supported by specific studies [55]. There is also no uniform definition of suppressed renin or elevated aldosterone concentration. A suppressed (or very low) plasma renin activity or renin concentration (particularly in the presence of interfering drugs known to stimulates renin release) can be defined as less than 0.6 ng/mL/hr or less than 5 mU/L. In the presence of suppressed renin, an aldosterone concentration >15 ng/dL or >416 pmol/L could be considered “inappropriately” elevated.

A 24 h urine collection for sodium and potassium can also be used to help the interpretation of ARR and corroborating a diagnosis of PA. A high sodium diet lowers renin more than aldosterone, potentially leading to false-positive results. On the contrary, low sodium diet increases plasma renin, and to a lesser extent, aldosterone levels, leading to false-negative ARR results (increasing the risk of misinterpreting milder cases of primary aldosteronism). Moreover, an inappropriately high 24-h urinary potassium excretion (for example greater than 30 mmol/24-h in a patient with sustained hypokalaemia) is highly suggestive of aldosterone excess at the level of the distal nephron.

**Fig. 2:**
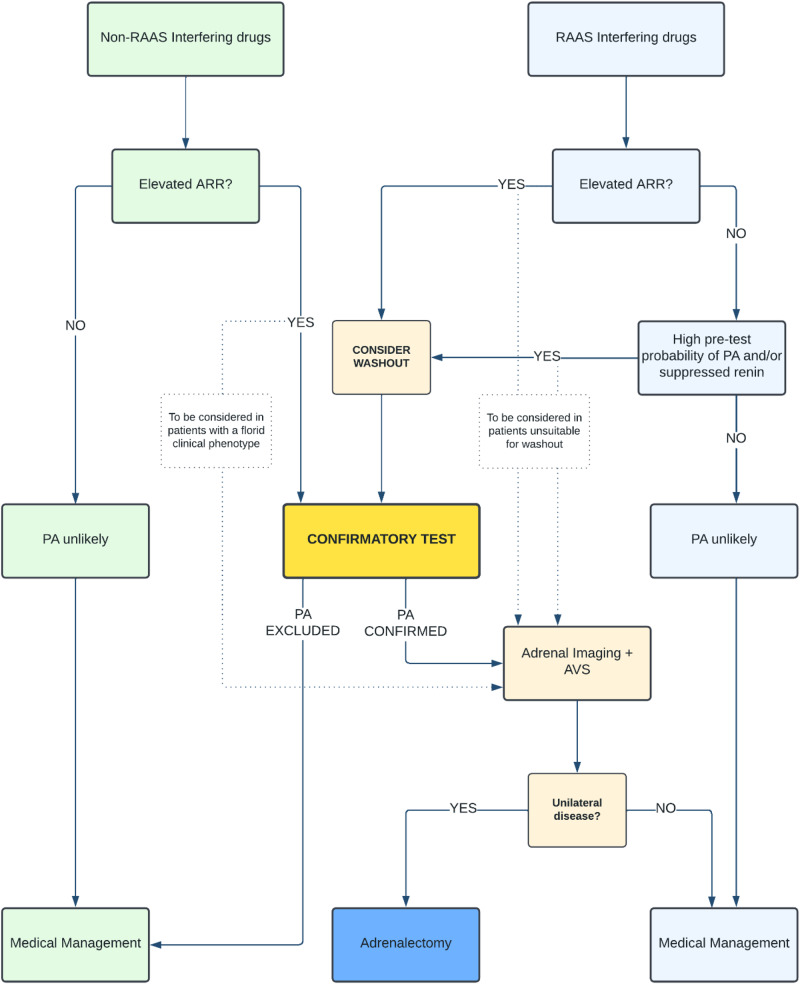
Simplified BIHS algorithm for the diagnostic workup of suspected primary hyperaldosteronism (PA) in patients with arterial hypertension. PA primary hyperaldosteronism, ARR aldosterone renin ratio, AVS adrenal vein sampling.

Based on these premises, we propose a pragmatic diagnostic approach (Fig. 2) to increase the detection rate and to maximize the opportunity to initiate target treatment.

1. Before evaluating ARR, consider discontinuing β-adrenergic antagonists and centrally-acting drugs (such as clonidine and alpha-methyldopa), which suppress renin release and increase the ratio of false positive results (Table 2). Do not routinely discontinue all the other antihypertensive medications but screen for PA at the time of the patient encounter since knowing the effects of different drug classes on ARR and its components can assist in the decision-making process [56–59].
2. If subjects are on treatment with non-RAAS interfering drugs (such as long-acting non-dihydropyridine calcium channel blockers, doxazosin and/or moxonidine) interpret the ARR using the cut-off values proposed in Table 3 [1]. Cut off values might differ among local laboratories and standard operating procedures should be taken into account. Of note, an optimal cut-off value of ARR for the screening of PA is not universally agreed and some authors have suggested that with aldosterone measured in ng/dl and renin in mUI /L, an ARR cut-off greater than 2.06 has a high overall accuracy (sensitivity and specificity) for the identification [54].
3. If subjects are on other classes of antihypertensive medications, consider the individual components of the ARR.
4. If the ARR is elevated and/or renin is suppressed despite the interfering effects of medications (excluding β-adrenergic blockers and centrally-acting drugs), diagnostic workup of low-renin hypertension should include PA (Fig. 2).
5. If renin is not suppressed and ARR is not elevated, PA is unlikely. However, ACE-I, ARBs and diuretics have the potential to elevate renin in patients with mild PA. Therefore, the finding of non-suppressed renin in a patient taking those drugs does not completely rule out the diagnosis of PA and clinical characteristics of the patient should be considered.
6. In patient with high pre-test probability of PA (such as with hypokalaemia and/or resistant hypertension,) pharmacological washout and/or adrenal imaging/confirmatory tests should be considered.

### Overall summary of recommendations

- Use plasma aldosterone to renin ratio (ARR) as screening test for PA.
- Consider discontinuing β-adrenergic blockers and centrally acting drugs before performing ARR.
- Withdrawal of all antihypertensive medications for ARR screening is not routinely recommended.
- When assessing ARR consider confounding factors such as sodium intake, hypokalaemia and drug treatment.
- Interpret the ARR using the cut off values in Table 3, unless subjects are on drugs that affect the renin-angiotensin system (RAAS), in which case consider the individual components of the ARR—e.g a normal ARR but suppressed renin whilst on drugs expected to increase renin does not exclude PA.

## Case confirmation

Most guidelines/expert opinions suggest performing one or more confirmatory tests to definitively confirm the diagnosis of PA. These tests are based on the premise that in PA the aldosterone secretion is at least in partially autonomous from renin and therefore angiotensin release [60]. Tests are also justified by the need to avoid unnecessary invasive procedures such as adrenal vein sampling (AVS) or surgery. Commonly performed confirmatory tests comprise the oral sodium loading test, the saline infusion test and the captopril challenge test, and the fludrocortisone salt loading test (Table 4). Tests should be performed according to local expertise and availability and usually require a careful preparation of the patient. In centres where dedicated facilities are not available, oral sodium loading test and captopril challenge tests should be considered since they can be performed in an outpatient setting. Despite the importance usually attributed to confirmatory tests, their use is not evidence-based [61–64], as none of the studies supporting their use for diagnosing primary aldosteronism met state-of-the-art criteria for validation of diagnostic tests [3].

**Table 4:** Confirmatory tests used for PA diagnosis.

Test	Saline suppression tests	Oral salt loading	Fludrocortisone suppression test	Captopril challenge test
**Procedure**	Venous infusion of 2 L of saline solution in 4 hours (seated position preferred^104^)	Oral intake of 6 g/day sodium chloride for 3 days and potassium chloride to correct hypokalemia. 24-h urinary sodium (urinary sodium >200 mEq/24 h) U-Aldo sampling from day 3 at 08:00 h until day 4 at 08:00 h	Oral Intake of 0.1 mg/6 h fludrocortisone for 4 days, potassium chloride and sodium chloride (6 g/day). Blood sampling for plasma potassium/6-h (potassium >4 mmol/L) and 24-h urinary sodium from day 3 to day 4 (>200 mEq/24 h) On day 4, plasma cortisol at 07:00 h and ARR and plasma cortisol are at 10:00 h	Oral intake of 25–50 mg captopril after 1 h in seated position. Blood sampling for ARR at baseline and after 2 hours
**Interpretation**	PAC not suppressed after saline infusion (> 6.8 ng/dL (190 pmol/L) proposed as the most accurate threshold [65,105–107])	U-Aldo <10 µg/24 h (28 nmol/24 h) excludes PA [61]. It has been proposed that U-Aldo >12 or 14 µg/24 h (33 or 39 nmol/24 h) could be used as cut off to confirms PA	PAC >6 ng/dL (170 pmol/L) confirms PA Test validity if PRA <1 ng/mL/h and plasma cortisol 10:00 h < cortisol 07:00 h [1, 64]	After 2 hours PAC decrease <30% and suppressed renin confirms PA [1]. Proposed threshold ARR > 30 ng/dL or PAC > 8 ng/dL after challenge.
**Advantage**	Relatively inexpensive and widely used internationally	Relatively inexpensive and can be performed in outpatient setting. The sensitivity and specificity of the oral sodium loading test are 96% and 93%, respectively^111^.	Regarded by some authors as the most accurate confirmatory test for the demonstration of angiotensin II independency of aldosterone secretion	Relatively inexpensive and can be performed in outpatient setting
**Disadvantage**	To be performed in a medical facility with trained personnel. Hourly BP monitoring is recommended, and caution required in patients with severe kidney disease and/or heart failure	Its reliability depends on the accuracy of 24-h urine collection Contraindicated if severe hypertension, kidney failure, cardiac arrhythmia, severe hypokalemia.	Expensive and labor intense. Because of risk of hypokalemia requires hospitalization	Risk of angioedema and contraindicated in case of suspected renovascular hypertension. `

*PAC* plasma aldosterone concentration, *U-Aldo* 24-hour urinary aldosterone, *ARR* aldosterone-renin-ratio, *PA* primary hyperaldosteronisms, *BP* blood pressure.

Since 2016 the Endocrine Society Guidelines, recognizing the burden (and perhaps limitations) carried by the confirmatory tests, suggested the possibility of bypassing them in patients with a florid clinical phenotype, e.g. an elevated ARR, high aldosterone concentration (>20 ng/dL) and spontaneous hypokalaemia [1]. The BIHS also supports the opportunity of considering avoiding confirmatory tests on a case by case basis in subjects unsuitable for washout who have elevated ARR and/or adrenal abnormalities (although this is not evidence based).

For patients who have had ARR measured without pharmacological washout from interfering drugs, it’s important to consider clinical features (such as difficulties in controlling hypertension or hypokalemia) alongside biochemical findings.

In order to reach a diagnosis of PA, the feasibility of pharmacological washout should be considered although is not always possible (particularly in patients with resistant hypertension and/or elevated cardiovascular risk). If that is the case, patients should have an adrenal imaging possibly including kidneys and renal vascular beds to exclude other secondary forms of hypertension. Alongside the adrenal imaging a 24 h urine collection for aldosterone excretion could also be used to corroborate the diagnosis. In case of suppressed renin, an adrenal lesion and a urinary aldosterone excretion >10 mcg/24 h (with a urinary sodium excretion >200 mEq/24 h) patient might be considered candidates for to AVS. Otherwise, the opportunity of considering surgery, performing AVS or arranging further confirmatory tests without extensive switching of antihypertensive treatment [65] should be evaluated on a case by case basis (Fig. 2).

### Overall summary of recommendation

- A confirmatory test performed off drugs affecting the RAAS is routinely required to support a diagnosis of PA (Table 4).
- In patients not suitable for pharmacological washout the need for confirmatory tests should be evaluated on a case-by-case basis (Fig. 2).
- In patients with a florid PA phenotype (clearly elevated ARR, elevated aldosterone concentration and hypokalaemia) confirmatory tests can be omitted.

## Subtype evaluation

An imaging test is recommended in all patients with suspected PA [1] although absence of morphological of adrenal abnormalities should not exclude patients from potential surgical treatment [66]. Discrepancy between imaging findings and AVS is relatively common [67, 68]. High resolution computed tomography (HRCT) and magnetic resonance imaging (MRI) are commonly used to examine adrenal lesions in both symptomatic and asymptomatic patients. In centres with radiological expertise, MRI should be considered the initial imaging modality given the good specificity for detection of lipid rich adenomas and lack of ionizing radiation. Both HRCT and MRI have limitations in detecting micro-adenomas and poor accuracy in predicting unilateral disease.

To identify candidates suitable for unilateral adrenalectomy, AVS is recommended [1, 4]. AVS should be offered only to patients who a) have evidence of PA b) are willing to consider adrenalectomy and c) have no major contraindications to the intervention. AVS requires considerable experience in its execution and interpretation, and should be performed in specialist referral centres [69] following accurate preparation of the patients. Despite some suggestions that AVS after ACTH stimulation can reduce the number of unsuccessful cannulations [70] some authors have questioned its utility and local expertise should be used., Specialist review of the AVS and a multidisciplinary discussion including a hypertension specialist, imaging specialist, and surgeon is advisable. A unilateral form of PA can be identified in more than half of cases when AVS is used systematically [55].

### Overall summary of recommendation

- All subjects with confirmed PA should have adrenal imaging (MRI/HRCT).
- Morphologically normal adrenal glands do not exclude PA.
- Adrenal vein sampling (AVS) should be performed to identify potential candidates for unilateral adrenalectomy.

## Treatment

Despite the absence of randomized controlled trials (RCTs) comparing outcomes between adrenalectomy and medical management in unilateral PA, observational studies have shown the efficacy of the surgery in reducing cardiovascular events [37, 71–75]. In case of unilateral disease, laparoscopic adrenalectomy should therefore be considered the preferred treatment option in subjects willing to consider surgery. Predictors of success (in terms of cure of hypertension) include young age, female sex, a short history of hypertension, a high number of antihypertensive medications requirement, an absence of vascular remodelling, and/or renal chronic kidney disease [76].

Biochemical cure (i.e., normalization of plasma aldosterone levels) is achieved in the majority of all patients following adrenalectomy [77]. However, results on clinical cure (such as postoperative normotensive state without the use of antihypertensive medications) vary extensively across studies (ranging from 22 to 84%) [77–80]. Even when AVS-guided, adrenalectomy has been reported to provide resolution of hypertension or marked improvement in blood pressure control only in a proportion of the treated subjects [81]. A long-term follow-up of 420 patients who had unilateral adrenalectomy for aldosterone producing adenoma in papers published from 1987 to 2001, showed that only approximately half of the subjects were improved or cured [82]. Even lower percentages after laparoscopic or open adrenalectomy were reported from a small cohort at the Mayo Clinic [83]. Thus, from the hypertension perspective, normalization of hyperaldosteronism and treatment of hypokalaemia after adrenalectomy does not always lead to normalization/reduction of blood pressure values, suggesting that patient selection is still suboptimal [76]. Common reasons for failure to surgically cure PA are inaccurate diagnoses, i.e. non AVS-guided adrenalectomy, and/or, more frequently, the concurrence of chronic kidney disease, increased arterial stiffness and/or primary (essential) hypertension which involves up to 30% of the PA patients and cannot be expected to be cured by adrenalectomy alone.

If adrenalectomy is not clinically indicated or individuals are not willing to consider a surgical intervention, medical management should be initiated. MRAs, alone or in combination with other antihypertensive agents, are recommended in order to control blood pressure and achieve normokalaemia.

Spironolactone and eplerenone are MRAs available in the UK, although only spironolactone is licensed for primary hyperaldosteronism. Spironolactone, developed in the 1950s, has structural elements of the progesterone molecule; thus, it is associated with progestogenic and antiandrogenic adverse effects. Eplerenone is a spironolactone derivative designed to enhance selective binding to the mineralocorticoid receptor, whilst minimizing binding to progesterone and androgen receptors [84]. MRAs-induced side effects are common, dose-dependent, and can be troublesome particularly in men [85]. Although eplerenone is more selective (thus potentially causing fewer side effects), it is also a less potent antagonist of the MR, and shorter acting, compared with spironolactone [86]. In general, eplerenone must be dosed twice as high as spironolactone for therapeutic equivalence [87]. Clinical evidence suggest that when used at an appropriate dose, both drugs are safe and effective treatment in patients with PA [88].

Spironolactone has a slow onset of action relative to vasodilators and is usually started at a relatively low dose (12.5 - 25 mg once daily). Electrolytes, creatinine, and blood pressure should be reassessed within 4 weeks (before titrating the dose upward), but these tests could be assessed sooner in patients with renal insufficiency who are prone to develop electrolytes abnormalities since the risk of severe hyperkalaemia is mitigated by appropriate monitoring [89]. Because PA induces a hyperfiltration state, a small rise in serum creatinine is expected in PA patients on treatment with MRA, reflecting the effects of the drug on glomerular hemodynamic. Spironolactone can be increased to the maximum tolerated dose until the patient maintains normokalaemia without potassium supplementation (usually achieved with doses of spironolactone <100 mg od). Non-adherence and incomplete adherence are common in resistant hypertension [90] and should be evaluated particularly in subjects with refractory hypokalaemia despite MRA treatment. Doses of spironolactone >100 mg daily for PA have reportedly been used [90] with anecdotal reports of 200–400 mg daily doses used chronically for PA [91]. Plasma renin might be used as an additional marker to evaluate successful aldosterone blockade [75, 92]. In cases of persistent renin suppression, increasing the MRA dose should be considered, provided that there are no contraindications (e.g., antiandrogen side effects, hyperkalaemia, reduced kidney function). Common side effects of spironolactone in women include spotting and breast tenderness; contraception for those of reproductive potential should always be advised. Men can develop gynecomastia and sexual dysfunction due to spironolactone’s antagonism of testosterone. The incidence of gynaecomastia may be as high as 30% at 100 mg daily and 62% at 200 mg daily [93]. If adverse effects to spironolactone develop, eplerenone can be considered an off-label substitute, usually given twice daily at a dose twice that of spironolactone.

An adjunct to MRA treatment is dietary sodium restriction [1], which helps to reduce urinary potassium loss. Particularly in patients on treatment with thiazide and thiazide-like diuretics, the epithelial sodium channel inhibitor, amiloride, is also a valuable strategy to control blood pressure and assure normokalaemia in case of MRA intolerance. Combination of an MRA and amiloride have been used anecdotally but requires monitoring of kidney function and potassium levels. Although amiloride may have a role in controlling blood pressure and increasing the potassium level [94, 95] its use in patients with PA is anecdotal [96, 97].

Despite the fact that medical management of PA is usually considered effective in controlling blood pressure, uncertainty remains in how adequately the medical therapy counterbalances the effect of the neurohormonal activation in PA. In this respect, one large cohort study of PA patients treated medically or surgically found a higher incidence of cardiovascular mortality among those treated medically [75]. In that study however, selection bias could not be excluded. Until more data from prospective studies are available, surgical therapy should be the preferred option in treatable cases in patients willing to consider surgery [98].

### Overall summary of recommendation

- Adrenalectomy should be considered in unilateral PA.
- In patients who are not suitable for, or do not wish to have, an adrenalectomy, mineralocorticoid receptor antagonists (MRAs) alone, or in combination with other antihypertensive drugs, are recommended.
- Spironolactone is the first-line MRA.
- Consider eplerenone in patients with contraindications or intolerance to spironolactone (NB spironolactone 25 mg/day is equivalent to eplerenone 25 mg/twice a day).
- Amiloride (5–40 mg/day) may used as a substitute for, or in addition to MRAs, particularly in patients intolerant to MRAs or not at blood pressure target.
- Monitor electrolytes, creatinine, and blood pressure response during treatment with MRAs.
- Up-titrate MRAs to the maximum tolerated dose to achieve normokalaemia and blood pressure control. If this cannot be obtained additional medication may be required.
- Dietary sodium restriction should be encouraged alongside MRA treatment.

## Future perspectives

A better understanding of the disease and the factors affecting outcomes will better inform selection of either adrenalectomy or medical management. The identification of “subclinical aldosteronism” [12, 99] also raises more questions regarding the natural history of the disease but more importantly regarding the causal relationship between aldosterone and blood pressure response. The clinical significance of elevated ARR in non-Caucasian patients has also not been extensively evaluated and the prevalence of PA in subjects with low renin hypertension has not been assessed.

The current clinical pathway for the diagnosis and management of PA poses multiple complexities and burdens onto healthcare professionals and patients. To improve this situation novel biomarkers with better sensitivity and specificity and a simpler, safe confirmatory test are needed as well as an alternative to AVS. Amongst such tests, C_11_-Metomidate PET-CT scanning has been proposed as an adjunct or alternative to AVS but has limited availability and still requires further large-scale assessment [100, 101]. Several prediction scores developed have however failed to provide similar validity as AVS. Newer imaging techniques with or without combination of using molecular markers have generated great interest, though currently their use is limited to research due to costs, availability, and validation [102]. The genetic basis of the various somatic mutations detected are expected to lead to the development of personalised therapies [103]. Finally, development of newer therapeutic agents with specificity to the mineralocorticoid receptor and selective aldosterone synthase inhibitors might lead to efficacious and safer therapy options. To enable these above objectives a better data infrastructure such as development of a robust registry, to collate extensive real-world evidence might be the best way forward.
